# Balancing the load: A narrative review with methodological implications of compensatory training strategies for non-starting soccer players

**DOI:** 10.5114/biolsport.2024.139071

**Published:** 2024-04-25

**Authors:** Filipe Manuel Clemente, Guglielmo Pillitteri, Luiz H. Palucci Vieira, Alireza Rabbani, Piotr Zmijewski, Marco Beato

**Affiliations:** 1 Escola Superior Desporto e Lazer, Instituto Politécnico de Viana do Castelo, Rua Escola Industrial e Comercial de Nun’Álvares, 4900-347 Viana do Castelo, Portugal; 2 Sport Physical Activity and Health Research & Innovation Center, 4960-320 Viana do Castelo, Portugal; 3 Gdansk University of Physical Education and Sport, 80-336 Gdańsk, Poland; 4 Sport and Exercise Sciences Research Unit, Department of Psychology, Educational Science and Human Movement, University of Palermo, Palermo, Italy; 5 PhD Program in Health Promotion and Cognitive Sciences, University of Palermo, Palermo, Italy; 6 Universidad César Vallejo (UCV), Facultad de Ingeniería y Arquitectura, Escuela Profesional de Ingeniería Industrial, Grupo de investigación en Tecnología aplicada a Seguridad ocupacional, Desempeño y Calidad de vida (GiTaSyC), Campus Callao, 07001 Lima, Perú; 7 Sport Science Department, Ittihad Kalba F.C, UAE; 8 Sport Science Department, OFI Crete F.C, Crete, Greece; 9 Jozef Pilsudski University of Physical Education in Warsaw, Warsaw, Poland; 10 School of Health and Sports Sciences, University of Suffolk, Ipswich, UK

**Keywords:** Football, Top-up, Sports training, High-intensity interval training

## Abstract

New training approaches have emerged advocating for the implementation of compensatory physical training. This approach aims to provide additional training that balances the load typically experienced by non-starters during a match. This may help maintain their readiness and ensures that their physical fitness is not compromised by the reduced exposure to match loads. Thus, this narrative review aims to describe the differences in external loads between starting and non-starting players and describe the studies conducted in compensatory training. Studies examining external load metrics such as total distances covered, high-speed running, and sprinting suggest that, adjusted for playing time, values are often higher in non-starting players. Although not standardized, there is an obvious decrease in exposure for these critical variables in non-starters. Additionally, internal load parameters such as perceived exertion and heart rate tend to be higher in starting players. Regarding the physical fitness impacts, evidence suggests differences observed between starters and non-starters in some aspects of physical performance, although the extent and significance of these differences can vary. The studies on compensatory training are limited, and the typical approach usually centres on running-based exercises and small-sided games, offering differing approaches to address the physical needs. The gap in research underscores the necessity for improved study designs that can shed light on the real impact of compensatory training. Presently, the practice of compensatory training has been adopted, yet a definitive understanding of its genuine influence, particularly in terms of enhancing physical fitness and mitigating injury risks, remains elusive.

## INTRODUCTION

Research focusing on the disparities in training and match loads between non-starter players and starters has been steadily increasing over the years. A very basic search on PubMed using the terms (“non-starter*” AND (soccer OR “football*”)) yielded results indicating that publications since 2021 constitute 48% of the evidence available on this topic as of February 9, 2024.

Non-starter players, including substitute players and those not utilized during an official game, typically comprise those who are not chosen to participate from the commencement of a soccer match [1, 2]. According to regulations, the number of substitutions permitted in official elite soccer matches ranges from a maximum of three to five, depending on the specific rules of the federation, as outlined in the Laws of the Game for the 2023/2024 season [3]. These limitations result in non-starter players having a limited number of opportunities to partake in matches [4].

This situation implies that within the overall duration of a standard match – consisting of 90 minutes of regular playtime augmented by any supplementary time contingent upon the match’s unfolding (sometimes exceeding ten extra minutes) – certain players have little or no involvement [4, 5]. Match load denotes the combination of increased physical demands and intensity witnessed in soccer matches [6, 7]. Consequently, the absence of this relevant stimulus may lead to an inadequacy in load exposure for certain players [8]. Such a disparity may lead to an imbalance between those players who start matches and those who do not [2]. This could potentially result in a diminished capacity to conform to the anticipated chronic load [9] or to achieve the requisite physical fitness adaptation demanded at the level of competitive match play, especially in cases where non-starter players remain in such a role over a long period [10].

Given this unique circumstance, strength and conditioning coaches have begun to incorporate compensatory training methodologies [11]. These include running-based high-intensity interval training (HIIT) or small-sided games (SSG) [4, 12], intending to potentially rectify the load imbalance experienced by non-starting players. Consequently, soccer coaches and trainers, particularly in professional settings, have increasingly included supplementary ‘top-up’ conditioning sessions tailored for players who have remained unused or have only participated partially during official matches [4, 12].

Termed compensatory training or colloquially referred to as “top-up” training, these sessions are frequently administered within the post-match context – immediately following the conclusion of a match [13, 14]. Additionally, in alternative contexts, these supplementary sessions are scheduled for the day after the match (i.e., MD+1), synchronizing with the recovery phase of the players who started the match. In a survey encompassing 33 soccer coaches, it was revealed that 39% of respondents often devise training regimens and preparatory strategies tailored to non-match days for substitute players [4]. Meanwhile, 8% indicated that such strategies were occasionally employed, with an equivalent proportion employing them consistently [4].

Recognizing the paramount importance of ensuring equitable load exposure for non-starting players – akin to their counterparts who participate from the outset of a match – with the overarching goal of optimizing their ability to manage load and navigate diverse scenarios, the implementation of compensatory training emerges as a pivotal strategy [15, 16]. This strategy aims to equalize the chronic load of non-starting players, prevent detraining, and ensure the appropriate stimulus for enhancing physical performance improvements [17]. Additionally, it addresses the unique constraints imposed by soccer’s limitation on multiple substitutions, which sets it apart from other sports.

Despite the increasing popularity of compensatory training in soccer strength and conditioning practices, existing research in this area has not yet been systematically reviewed. This often leads to sporadic selection of articles, failing to provide an overview and summarized evidence necessary for identifying gaps and aiding practitioners in refining their practices. Given the emergent nature of this topic in recent years, a narrative review can offer interpretation, critique, and significant contributions to deepening understanding [18]. Such an approach may shed light on the current state of the field and uncover underexplored topics for further investigation.

Considering this context and opportunity, the present narrative review aims to elucidate compensatory training studies while providing an overview of the current state of research regarding comparisons between starters and non-starters in terms of match load, training load, and physical fitness differences. In addition to describing existing studies, this review seeks to identify research gaps and offer a practical example of compensatory training implementation based on available literature findings.

### Distinguishing factors between starting and non-starting players in soccer

When examining differences between starters and non-starters, the studies primarily focus on two main dimensions [5, 19, 20]. One dimension pertains to the exposure of both groups to regular match loads, which ultimately may affect the total weekly load accumulated [2, 19, 20]. As a potentially related factor, these disparities may influence physical fitness adaptations to some extent [21]. Therefore, both topics are worthy of description, with the aim of identifying what the literature describes regarding differences between starters and non-starters.

### Match load disparities between starting and non-starting players in soccer

The total distance covered (TD) is a traditional metric of external load and was generally greater in starting as compared to non-starting players in studies including senior male [22–25], female [26, 27] or youth (male) athletes [28]. Divergent results were found only in a separate study combining data (sum) of replacement and replaced players [29] and another one using only friendly matches [30]. In these cases, male senior starting athletes showed lower in-game TD or no differences as compared to non-starting peers. In studies taking in-game TD standardized by playing time, contrasting results to those reported for total TD were observed; higher values of standardized TD were reported for non-starting male youth [31, 32] or senior players [23, 33–40], in a total of 11 studies, while two demonstrated no differences [22, 41]. In female seniors, the results are not yet conclusive, because the two existing studies reported conflicting evidence of no between-status differences [42] or higher values in non-starting athletes [43].

Concerning high-speed running (HSR) outputs, mixed results were identified for the total amount expressed in male senior players, i.e. starting [24, 25, 40] and non-starting [29, 30, 44] having superior distances covered to their peers in three studies each. On the other hand, in-game HSR distance standardized by playing time was consistently reported to be higher in non-starting athletes [33–39, 41, 45]. Similar findings were identified in youth, in which no between-status difference [28] or higher values in starters [46] occurred simultaneously when taking absolute HSR data while standardization by playing time revealed superior distances at HSR in non-starters [31, 32]. In female senior studies indicated higher TD at HSR in starters [26, 27]; when adjusting by playing time one study showed higher values in non-starters [43] and another no between-status differences [42].

In the sprinting domain, there is an equivalent number of studies which found higher total values in starters [23–25] and no between-status differences or higher values in non-starters [29, 30, 44]. Conversely, there was a general tendency for non-starting players to experience greater standardized – by playing time – sprinting activity, i,e. six studies [35, 37–39, 41, 45] versus two showing similar results among statuses [22, 34]. In male youth players, no consensus was reached regarding whether total sprinting is dependent on player status [28, 46] while no studies were found reporting standardized sprinting data. For female senior players, the results of sprinting activity (total and standardized) were in line with those of HSR mentioned in the previous paragraph.

Peak demands – or worst-case scenarios (WCS) – represent a type of metric that has gained popularity over recent years and was also appraised. In this sense, peak game speed (also known as in-game maximal sprinting speed) was demonstrated most frequently to be faster in starting male senior players as compared to non-starting peers [35, 37–39]. The exceptions showing no differences were the same as in the case of total TD [29, 30], in other words, derived from observations of only friendly events or official ones yet calculating peak speed as the highest value between players involved in a substitution (pooling the starting/replaced and non-starting/replacement players). Regarding the WCS for TD, mixed results of being greater [47] or lower [48] in starting players were simultaneously identified, with also only a trend towards differences between status in WCS for HSR and sprinting. However, additional studies are needed to draw firm conclusions.

From the perspective of internal load parameters, match-derived ratings of perceived exertion were consistently greater in starting as compared to non-starting male (youth/senior) and female players [27, 30–32, 4–51] and the same seems valid for heart rate indices [25, 46]. Finally, across the aforementioned studies reporting ingame internal and/or external load measures, exposure – playing time – of non-starting male and female senior players ranged respectively from 13 ± 6 [38] to 25 ± 7 minutes [36] and 22 ± 13 [43] to 36 ± 14 minutes [26]. In young male soccer players playing time ranged from 23 ± 13 [50] to 50 ± 7 minutes [28], while there were no reports for young female soccer players among studies examined here.

### Physicalfitness disparities between starting and non-starting players in soccer

A high fitness level can be considered an important condition to allow soccer players to withstand the match demand characterized by high intensity actions interposed by lower intensity activities. The unbalanced external load exposure during official matches between starter and non-starter players could determine less physical fitness conditioning for players with inadequate play time or adjustment in physical training such as compensatory training (e.g., intermittent running, sprint training or SSG).

Aerobic fitness has been considered one of the most important physical variables at a high soccer level [52, 53]. Different markers such as maximal oxygen consumption ($\dot{\text{V}}$O_2max_), have been related to competitive ranking or team level [54]. Worth noting, in-season fixture congestion may reduce the opportunities for implementing physical training that could progressively lead to aerobic fitness deterioration in the long term.

Sporis and colleagues [55] found that official match play time was related to maintenance of physical fitness in professional soccer, while in another study [21] no individual negative change occurred for the non-starters in terms of $\dot{\text{V}}$O_2max_ compared to starters who had a more pronounced aerobic decrement. Moreover, the same authors reported that the mean team velocity at maximal oxygen uptake was not statistically significantly different between July and December for both starter and non-starter elite male soccer players [21]. A study [56] carried out on collegiate female soccer players reported no significant differences in physical ability between non-starters and starters by assessing 30-m sprint time, pro-agility test, and Yo-Yo intermittent recovery test level 1 (YYIR1) scores. Similarly, a study [57] compared the physical features and performances of Japanese elite female soccer players and found no significant difference in physical performance between starters and non-starters.

Conversely, the authors observed a higher value in starters in terms of the maximum isokinetic concentric strength performed by fast contractions such as an angular velocity of 300°/s compared to non-starters [57]. This result indicates a greater power level for the players most involved in official matches. The ability to develop actions at high intensity is related to the ability to integrate speed and changes of direction, assessed by very high-intensity running and sprint distance [58]. For this reason, a study [19] that involved a Colombian youth women’s soccer team analysed the relationship of the physical variables of the squat jump, counter movement jump, counter movement with arms, right leg-left leg asymmetry, hamstring strength, change of direction, and speed in 5, 10, and 15 m as an influence of being a starter and non-starter. The authors found no differences in strength capacities and change of direction between starters and non-starters [19]. These results show that the starting and non-starting players present a similar physical performance evaluated based on strength, asymmetries, hamstring strength, change of direction, and speed.

Given the limited amount of research on this matter as in the cases of Colombian females [19] and Japanese females [57] or in male soccer players [21], it appears that whether one is a starter or a nonstarter does not significantly influence physical fitness levels. As a result, the hypothesis regarding the impact of this status remains unsubstantiated, albeit due to constraints in research quantity and diversity. Moreover, the extent of evidence regarding its impact on other aspects such as tactical and technical abilities, as well as complementary factors like body composition, remains largely unknown and insufficiently explored.

However, several factors such as tactical requirements, players’ response, playing position, fitness level and individual characteristics are related to physical performance [59]. The literature has also focused on body composition and its implications on physical performance [60]. Indeed, a study [60] examined the relationship between body composition and tests of speed, power, and cardiorespiratory fitness in male collegiate (Division I) starter and non-starter soccer players. The authors found that the difference between starters and non-starters for height was less than 1 cm and for body mass was less than 0.1 kg [60]. These results agree with other studies carried out in Norwegian championships (41) and England International players (< 16 years).

### The significance of high-speed running and sprinting training for establishing robust conditioning in soccer players: concerns of underexposure for non-starting players

When comparing the differences in external loads between starters and non-starters, it is imperative to consider key variables such as HSR and sprinting [61]. These activities involve substantial mechanical loads that are crucial for physical resilience and the ability to endure the demands of a match [62]. Notably, HSR and sprinting are predominantly observed during matches rather than training sessions [63], with minimal occurrence in training [64]. Therefore, particular attention should be paid to exposing players to near-maximal efforts, as some research suggests that programming > 95% of maximal sprint speed exposures may help reduce the incidence of hamstring injuries during elite soccer matches [65]. Consequently, the absence of match exposure may result in a significant deficiency that cannot be adequately compensated for during regular on-field sessions, particularly without specific considerations for non-starting players.

During official matches, HSR and sprint running distances ranged from 618 to 1,001 m and 153 to 295 m, respectively, in professional male soccer players. This amount of high-intensity running is performed by starter players only while non-starters need to be subjected to a similar amount of training during the following micro-cycle to avoid possible physical decrements.

The role that HSR and sprinting training play in the development of physical capabilities, sport-specific performance and injury prevention among soccer players has been described in detail [62]. However, the monitoring and quantification of running intensities have been frequently debated without finding a definitive agreement. Specifically, Gualtieri et al. [61] stated that there is no consensus on the absolute thresholds defining HSR and sprint running in adult soccer players. Men’s and women’s soccer players use a range of speed thresholds that are frequently defined with the same term (e.g., HSR and sprinting distances) but correspond to different speeds, which complicates the comparison among teams and studies. Anyway, traditionally, absolute thresholds for HSR and sprinting distance have been set at 19.8 km · h^-1^ and 25.2 km · h^-1^, respectively [62]. Another approach used by practitioners consists of quantifying velocity thresholds using a relative approach, where the intensity is calculated as a percentage (e.g., 80 or 90%) of the peak speed [66, 67].

This approach allows for consideration of inter-individual players’ differences during training load monitoring and can have important implications for specific training sessions whose goal is to reach nearmaximal velocity exposure [61]. Furthermore, a recently published scoping review explored load quantification using both absolute and individualized running speed thresholds in team sports. The authors reported that thirty-four articles used individualized speed running thresholds based on physical fitness or performance assessments (e.g., 40-m linear sprint). However, some other physiological approaches (e.g., percentage of the maximum aerobic speed deriving from a physiological test – $\dot{\text{V}}$O_2max_ test) can also be used to quantify training intensity [61]. However, the non-standardized use of intensity (speed) thresholds in soccer can create some issues for practitioners.

For instance, some of these thresholds derive from physiological tests that have been used in sports (e.g., endurance) that are drastically different from the physiological and energetic model of soccer [68], and therefore they could have limited relevance for it. Therefore, scientists and practitioners need to use individualized thresholds (deriving from physiological or performance assessments) that can help to more accurately quantify the training load of soccer players. This would facilitate the implementation of training protocols aiming to compensate the training load gap between starters and non-starters.

Based on such evidence, it is clear that practitioners need to put in place some strategies to “top up” the training load missed during the match by non-starter players (e.g., players who played < 30 min). Practitioners should design specific training drills that allow nonstarters to recreate in training the same intensities (distance/minute) and training stimuli needed to improve or at least maintain their sport-specific fitness level [69]. Beato et al. [69] reported that sided games with different formats (i.e., SSG, possession games, largesided games) can replicate and sometimes exceed some match-specific intensity parameters (e.g., number of accelerations and decelerations); however, HSR and sprinting distances were consistently lower compared to official matches.

Those findings were confirmed by another study that found that some sided game formats are more suitable for specific load-specific parameters than others; for instance, distance per minute, HSR, and sprinting exposure were greater during large-sided games compared to other, smaller formats, while the number of accelerations and decelerations was greater in medium-sided games compared to other formats [70]. The use of large-sided game formats to expose players to HSR demands is also supported by another recent systematic review [61]. The authors found that game-based drills designed in formats using relative areas per player greater than 225 m^2^ and 300 m^2^ appear to be adequate for achieving HSR and sprinting exposure, respectively [61]. Another valid training approach is based on soccer circuit-based drills, which offer some specific advantages to sided games (e.g., high load profiles and good reliability scores for both internal and external load parameters) [71], or on more traditional sprint running drills (without the use of the ball) [62]. Therefore, it seems that the combination of game-based, running exercises and soccer circuit-based drills is advisable to ensure adequate HSR and sprint running exposure at both a team and an individual level.

### Current research on compensatory training for non-starters: for whom, when, and how is it applied?

In recent years, strength and conditioning practices have undergone significant changes, incorporating compensatory training for nonstarters [4]. The rationale behind this shift is based on the belief that it provides the advantage of introducing a targeted level of training load [72]. This aids in sustaining the chronic load [2] while preventing imbalances during matches from exacerbating differences among starters. This increase in such practices has also sparked research interest in the field. Therefore, this section analyses how compensatory training has been implemented and explores potential findings related to this topic.

### Contextualizing compensatory trainingfor non-starters: exploring the research landscape and addressing current challenges

Recently, researchers have increasingly shown interest in exploring compensatory training for non-starters, as evidenced by the growing number of scholarly publications on the topicAn initial inquiry, conducted on PubMed and utilizing search terms such as “compensatory training,” “top up,” or “topup,” in combination with the keywords “football” or “soccer,” as of August 29, 2023, yielded a total of 10 relevant titles. Among these, 7 were published in the year 2022, while the remaining 3 were released within the current year of 2023. These statistics underscore the emergence of this subject as a burgeoning field of study, actively capturing the attention of sports researchers and practitioners alike.

At present, the majority of research related to compensatory training is concentrated on administering targeted external loads, predominantly focused on HSR distances. This specific external load measure is touted as integral in rendering players resilient to manage match intensities and loads, while concurrently bolstering their capacity to mitigate injury risks.

One of the challenges inherent in compensatory training is determining the appropriate dosage. For instance, depending on the extent of a substitute’s participation in a match, the dosage of a prescribed HIIT regimen would need to be adjusted accordingly. Furthermore, challenges arise when compensatory training is not immediately introduced after a match, but rather during the preparation for the next match.

An optimal window for such training might be the training session immediately following the match day, as this typically coincides with a restorative training routine for the starting players [73]. However, integrating compensatory training on days beyond match day + 1 proves to be more complex. The structural adjustments necessary for such sessions, coupled with the logistical issues arising from individualized comparisons to starting players, present formidable challenges for coaches. Additionally, introducing an additional training dose on such days could also impact the overall adaptation to the training load for the week, potentially adversely affecting player physical performance over that period.

The considerations surrounding tailored physical training for nonstarting players remain an ongoing discourse. As revealed in a recent survey [4], 39% of coaches frequently devise specialized training for non-starters on non-match days, while 31% reported occasional implementation, and 15% acknowledged that such preparation is virtually absent. Among the factors contributing to the infrequent implementation of such training programmes are uncertainties stemming from unknown substitute playtime during matches and logistical intricacies concerning training session structuring or squad announcements within a timely manner [4].

If these preceding responses pertain to the planning of compensatory training for the day after a match, the immediate training session following a match also presents its challenges. One such challenge arises from the compressed schedule of matches, which leaves little leeway for additional training, especially considering the team’s return travel commitments. Moreover, crafting an individualized training stimulus becomes imperative to ensure effectiveness. This implies the need for a streamlined process that accommodates the player who commenced the second half as a substitute in contrast to the player who entered the game during its final three minutes.

Furthermore, regulating the dosage of training, particularly in terms of metrics such as HSR, or sprint distance, poses its own set of complexities. The absence of a consensus or conclusive evidence delineating the precise extent of training a player should undergo relative to their typical in-game demands adds to the challenge.

### Characterizing acute responses to compensatory training in nonstarter players

In an endeavour to elucidate the post-match compensatory strategies for substitute players, a comprehensive study encompassing 37 matches and involving 31 professional United Kingdom premier league soccer players was undertaken [12]. The findings of this study revealed that the average duration of compensatory training was 17.13 ± 7.44 minutes, enabling an approximate coverage of 1.7 ± 6.2 km and 0.4 ± 1.7 km in distance covered and HSR distance, respectively [12]. The variability in the imposed workload was contingent upon a multitude of contextual factors. These factors encompassed the player’s role as unused or substitute, the duration of their time on the field, the match’s location, outcome, time of day, stage of the season, and scheduling [12].

In another study focusing on post-match compensatory training, an exploration into the training regimen of the day following the match revealed distinct findings [74]. Specifically the study on professional players participating in the Spanish premier league investigated the extent of HSR (although under different speed thresholds: 21-24 km/h compared to the regular range of 19.8 to 24.9 km/h) [74]. The analysis indicated that substitutes who played 5 to 15 minutes recorded an average of 0.49 ± 0.31 km in HSR (running between 12 and 24 km/h). For those substitutes who participated for 15 to 30 minutes, the HSR distance averaged 0.96 ± 0.46 km. Similarly, substitutes egaged on the field for 30 to 45 minutes had an average HSR distance of 1.3 ± 0.54 km [74].

Upon analysis, it becomes evident that the observed average HSR distance falls considerably short of the typical range covered in a standard match, which typically ranges from 0.8 to 1.0 km over the course of a full 90-minute match [75, 76]. Taking into account that substitutes may cover approximately 0.1 km upon their entry into the match (naturally dependent on the match duration) [77], it becomes apparent that compensatory training still offers a lesser dosage than what would be expected to align with a typical full-match scenario.

Naturally, a concern arises regarding the pattern of dose application. While the HSR distance of 0.8-1.0 km is distributed over the span of 90 minutes during regular matches, for example the 0.4 km is accumulated within 17 minutes during the post-match compensatory training [12]. This pattern creates a pronounced peak in exposure for substitute players undergoing compensatory training, surpassing the regular range of 4.8 to 10.1 m per minute of HSR experienced in matches, with a significantly higher 28.1 m per minute covered in compensatory training. This aligns with the most demanding scenarios encountered during matches [78].

The implications stemming from load density remain incompletely studied, leaving various questions unanswered. For instance, should a 1-km HSR distance be executed with a high density of 30 m per minute? Should we consider the intensity (e.g., distance per minute)? Further research is warranted to investigate in more detail the intricacies of translating peak performance demands into meticulously tailored training prescriptions. This entails a specific focus on incorporating considerations of load density to facilitate more informed and effective training strategies.

The same query extends to another pivotal metric, namely sprinting distance. In the context of soccer, players often cover a sprinting distance (e.g., speed > 25.2 km/h) of up to 0.3 km throughout a full match [6, 79]. However, insights from a profiling study undertaken in the English Premier League revealed a notable contrast when it comes to compensatory training immediately after a match [12]. Specifically, these sessions led to a sprinting distance of only around 0.03 km, with the peak speed of approximately 7.0 m per second [12]. A parallel investigation conducted in Spain similarly noted that compensatory training conducted on the day after a match translated to an approximate sprinting distance of 0.04 km [74]. This indicates that sprinting in the context of compensatory training accumulates to roughly 10% of the TD covered during a full match.

### Exploring compensatory training strategies and their applications for non-starter players

While the predominant application of compensatory training has been embedded in running-based regimes, which brings forth inherent advantages such as customization, controlled load management, and swift dose administration (density), there exist alternative approaches worth considering. For instance, the incorporation of SSG – a subset of drill-based exercises that mirror simplified game scenarios while incorporating specific constraints to highlight targeted behaviours – has emerged as a prospective avenue to administer compensatory stimuli for non-starters.

In a noteworthy pilot study conducted among female professional soccer players from the Spanish first league who assumed nonstarter roles [11], a comparative examination of three distinct modes of compensatory training was undertaken. These encompassed running-based exercises, SSG, and a hybrid approach integrating both methods. The participants’ external loads were meticulously monitored, yielding insightful findings. Notably, when subjected to SSG or the hybrid regimen, non-starter players covered markedly shorter distances relative to the running-based exposure [11].

Furthermore, under the running-based intervention, the players exhibited notably increased high-intensity running and sprinting distances [11]. Regardless of the specific form of compensatory training adopted, the non-starter players consistently covered significantly fewer TDs compared to their starting counterparts during the match [11]. It is worth highlighting that non-starters who underwent the running-based intervention achieved non-significant values in terms of high-intensity running and sprinting – an interesting observation [11].

### Identifying gaps and proposing future research in compensatory training for non-starters

Considering the distinct nature of the research topic, there exists a limited body of knowledge concerning the potential effects of compensatory training on fostering adaptations in players who assume non-starter roles. This gap in research underscores the necessity for innovative study designs that can shed light on the tangible impact of compensatory training. Presently, the practice of compensatory training has been adopted, yet a definitive understanding of its genuine influence, particularly in terms of enhancing physical fitness and mitigating injury risks, remains elusive.

The causality and effectiveness of compensatory training are yet to be substantiated. At this juncture, its recommendation is largely rooted in observational studies and associated hypotheses regarding the correlations between training load, sustained fitness, and injury resilience.

Hence, the avenues for investigating compensatory training for non-starters diverge significantly and hold the potential to yield more comprehensive insights. For instance, evaluating the contrast between generalized and individualized compensatory training protocols is imperative. Equally important are determining the optimal dosage required, delineating the criteria guiding its formulation, and identifying the pivotal variables governing its prescription.

A thorough examination of the actual ramifications of implementing compensatory training for player readiness, performance sustainment or enhancement, and the potential for fostering resilience against injury risk is essential. These investigative pathways can be effectively bolstered through the exploration of dose-response relationships and tailored approaches. Parallel study designs that incorporate control groups allow meaningful comparisons to be drawn.

Such research holds the potential to expand our understanding of compensatory training’s true impact on non-starters and cultivating evidence-based recommendations to guide its practical application.

**FIG. 1:**
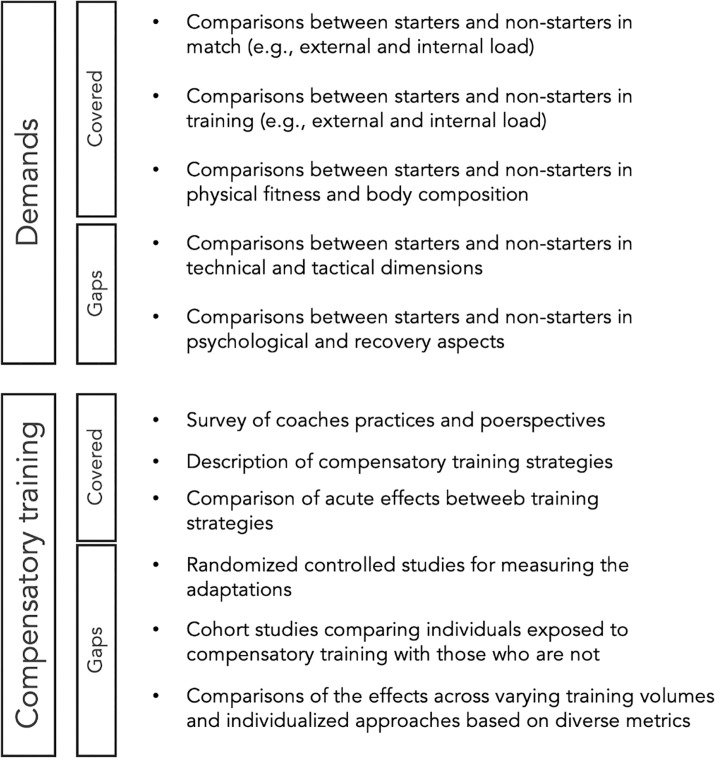
Overview of the topics addressed in the current state-of-the-art research on starter versus non-starter players and research gaps demanding further research.

Furthermore, when considering the comparison between starters and non-starters, it presents a significant opportunity for exploration. For instance, most studies are descriptive and tend to focus on a small number of teams. Additionally, there is inconsistency in describing the relationships between training and match loads, as well as in understanding the proportion of load volume across both situations. It would be valuable to conduct studies that integrate individualized training, incorporating the volume that non-starters miss from matches into compensatory training, and comparing this approach with those not exposed to such conditions. By integrating descriptive analyses of load differences between starters and non-starters and applying these findings to practice in both the short and long term, a more comprehensive understanding can be achieved (Figure 1).

### Practical approaches to implementing compensatory training for non-starter soccer players

**FIG. 2:**
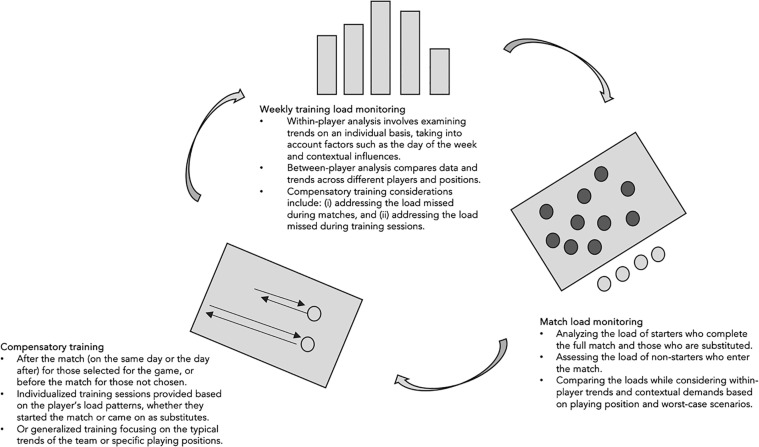
Potential aspects to explore when comparing starters versus non-starters.

Optimizing compensatory training is one of the most challenging tasks of sports science staff in soccer. However, in practice, it is more efficient to implement it via close collaboration with technical staff to ensure players train with the philosophy of the manager and achieve all possible physical, technical and tactical aspects simultaneously (Figure 2).

Indeed, in modern soccer fitness coaching, each manager has a game model and ideally all training details should be designed accordingly although with respect to the main principles of strength and conditioning [80, 81]. When designing compensatory training, the first factor to consider is the scenario or the weekly microcycle that players are approaching. For instance, compensatory training when implemented on MD+1 (match day +1) while MD+2 is off and the next match is after 5-6 days is completely different from when it is MD+2 (MD+1 is off) while it is also MD-2 for the next match. As there is a myriad of factors influencing such scenarios including match schedules, logistics, and manager preference to give a day off on MD+1 or MD+2, the first task of the strength and conditioning coach is to study the scenario and then try to design and implement the ideal possible compensatory session. As a logical rule, the closer the compensatory training is to the next match, the lower is the training level to prevent accumulated residual fatigue for the upcoming match as each non-starter player may be selected to play.

Although there is limited research in the area of compensatory training [11] and regardless of its complexity there is still no consensus agreement about its details, it seems that the main target should be to compensate external loads such as TD, HSR, sprinting, mechanical work and internal loads such as Edwards’ TRIMP and time spent in the red zone (> 85-90% of maximal heart rate) as much as possible considering the context [12]. It seems that the second target needs to be placed on special demands of the game (e.g., peak external or internal intensity, maximal velocity). However, due to a smaller number of players (typically < 10 outfield players) in compensatory training, achieving such a high amount of load in all factors is almost impossible and the ideal solution is to organize a friendly game with other teams or at least with younger teams in the same club. Research has shown that the smaller format of the game such as SSG (less than 4 v 4 + goalkeepers (Gks)) entails fewer external demands of the game especially in some important factors such as HSR distance [82, 83]. Organizing friendly games in compensatory training not only helps to achieve high and more balanced loads in all categories but also simulates the real scenario that players are being prepared for (i.e., the 10 v 10+Gk game) [84]

If organizing friendly games even with less volume (e.g., 2 x 30 minutes) is not possible due to logistics or a congested schedule in the weekly microcycle (e.g., MD+1 = MD-3), the strength and conditioning coach must first study the current manager style of the compensation training, the current typical load achieved, and the peak intensity and demands of the session to supplement it with special top-up solutions within or at the end of the session. For instance, if the priority of the manager is aggressivity and he implements man-to-man rules, which research has shown to increases the intensity [85], the strength and conditioning coach needs to know what current peak intensity might be achieved within the typical training session and then he needs to target/adjust more volume of external load measures to complement it. The other factors also include the number of players available and their individual fitness profiles. For instance, in one scenario there might be only four outfield players available due to other non-starters playing in the MD+1 for U21 or 2^nd^ team of the club and a strength and conditioning coach who normally uses 3 v 3+Gk or 4 v 4+Gks in compensatory training needs to find another solution to achieve his targets.

The use of medium-sided games (MSG) (5V5+Gks to 7 v 7+Gks) or particularly SSG in compensatory training is widespread thanks to their popularity and coaches’ interest in finishing the session with games. However, research has shown that implementing these formats although it inherently increases the intensity does not accumulate special and important demands of the real game (10 v 10+Gk) such as HSR distance [86]. The lower doses of these load-specific measures, in the long term, not only attenuate the fitness of players but also expose them to a higher risk of injury (e.g., HSR) due to the concept of acute to chronic load ratio [13]. To solve this problem, research suggests combining SSG and running-based HIIT [86] and many coaches supplement this MSG/SSG with analytical running exercises to top up and achieve higher doses of external loads [87]. In recent years, Bucchheit has developed an interesting method of supplementing training with running-based HIIT to address these issues [13]. Designing these HIIT methods via manipulating different influencing factors including the number of sets and series, straight running or running with the change of direction, the intensity and duration of work and rest periods, and the reference of defining the running intensity accordingly (e.g., V_IFT_, the maximum velocity achieved during the 30-15 Intermittent Fitness Test) can help to target not only external load demands (e.g., HSR vs. mechanical works) but also internal metabolic (aerobic vs. anaerobic) conditions [88]. However, strength and conditioning coaches need to be cautious and design/select special HIIT formats considering their special context and depending on other exercises of the compensatory training to avoid overload and increased risk of injury.

For instance, when compensatory training includes a high volume of an intense format of SSG (e.g., 2 v 2+Gk) which has already a high percentage of anaerobic metabolic contribution and the internal load is very high [89], it is more logical to supplement it with a neuromuscular format of HIIT (e.g., 8 reps of 10 s running with 110% of V_IFT_ with enough rest period between reps, 30 s) to avoid overload in the anaerobic metabolic part [90]. In contrast, when there is a congested schedule and MD is also MD-3 for the upcoming match and the player has not played, such a HIIT format can be adjusted with more volume of the work and greater contribution of anaerobic metabolic condition (e.g., 2 x 8 reps of 10 s running with 110% of V_IFT_ with 20-s rest in between reps and 1.5 min rest between sets).

To design these special HIIT formats, considering the training background, position and profile of the player is also important. For example, the volume of accumulated HSR distance is different for a winger vs. central back with profiles of almost 1000 m and 400 m of HSR distance, respectively [91]. Therefore, due to the complexity of designing compensatory training and supplementing it with different HIIT formats depending on a myriad of factors, here we present three scenarios with some examples to highlight the importance of first studying the context for strength and conditioning coaches (Table 1).

**TABLE 1:** Example scenarios of compensatory training sessions.

	Scenario A	Scenario B	Scenario C
Match Day	Sunday	Wednesday	Saturday
Next match	Sunday	Saturday	Thursday
Day off, if any	Tuesday MD+2	No day off	Sunday, MD+1
Compensatory session	Monday MD+1	Post-match & Thursday MD+1	Post-match & Monday MD+2
Available players for compensatory training	9 outfield player and 2 Gks are available. One player has played 30 minutes in the match and it was his first match after return to play from a hamstring injury, grade 2	7 outfield player and 2 Gks are available. One player (striker) has played 40 min but is young and very fit (he recovers fast)	7 outfield players are available from the bench and 3 players will join from out list players. The match is at home.
Manager Philosophy	Aggressivity and priority to play in deep with striker.	Fast decision making and quick transitions (attack to defence and defence to attack) behaviour	Possession of the ball and play wide with wingers
Compensatory training	-Jogging 10 min -Warm-up with passing drill 2 × 6 min -Ball possession 3 v 3+3 3 × 2 min + 30 s rest -4 reps of 30-m sprint with > 90% of maximal velocity with 45 s recovery in between reps -Game, 4 v 4+1 joker +Gks [20 × 40 m] Man to man, free touch -Running-based HIIT 1–2 × 8 reps of 10’’-20’’ with 110% of VIFT (the number of reps depending on player HSR profile)	-Post-match except the player who played 40 min other will do 1 × 8 reps of 15’’-15’’ with 90% of VIFT and 1 × 8 reps of 10’’-20’’ with 90% of VIFT. -In MD+1, -5 min jogging -Warmup with passing drill 2 × 3 min -Ball possession 3 v 3+1 joker 3 × 1.5 min + 1 min rest -2 reps of 30-m sprint with > 90% of maximal velocity with 45 s recovery in between reps -Game, 3 v 3+Gk [18 × 25 m] 6 × 2 min + 1.5 min rest	-After match will be short post-match training. This includes 10 min jogging, 5 v 5 ball possession for 4 × 3 min and 1.5 min rest in between, and finishing it with HIIT with the format of 12 reps of 10’’-20’’ with 110% of VIFT. -In MD+2, the compensatory training which is also MD-3 includes -5 min jogging -Warmup in with passing drill 2 × 4 min -Ball possession 3 v 3+1J+3 wall players 3 × 2 min (each 5 passes are counted as one point in possession) -2 reps of 30-m sprint with > 90% of maximal velocity with 45 s recovery in between reps -Game, 5 v 5+Gk [40 m × 32 m] 5 × 4 min with 1.5 min rest
Notes	In this example, the player returning from injury will have a modified session and plays as joker and does not do HIIT in the end as he is returning from injury and hamstrings are sore and not recovered well to expose to HSR. The coach philosophy is to play in deep and aggressively and therefore the length of SSG is double that of its width (40 vs. 20) and in order to increase intensity instead of touch limitation, the man to man rule is implemented.	-In this scenario to avoid accumulated fatigue in the next match which is very close, the load is split into two days to do running-based HIIT after the match and training with ball on MD+1 -The player who has played 40 min will train only until the end of ball possession and 10 min jogging at the end of the session -The rules in SSG include three touches maximum and middle line to pass condition for the goals being scored (vertical shift).	-In this scenario as the game is at home and MD+1 is off based on decision of manager, there is the possibility of training in stadium after the match and on MD+2. To address the philosophy of the manager the number of passes is counted with one coach for encouragement and the game is played in width pitch to play wider with wingers. Running-based HIIT also is in one long set (6 min) as there is one day rest following the game and enough time to recover for the next session (MD+2).

Gk: goalkeeper; MD: match day; HIIT: high-intensity interval training

As presented in Table 1, there are three main contextual factors when organizing compensatory training including match schedules, the number of players available and the manager game model and/ or philosophy. In the first scenario (scenario A), the match schedule is a normal weekly microcycle which has a day off on the second day (MD+2) and MD+1 is a good opportunity to train players with a very high load. In this scenario, the manager philosophy is playing deep with a striker and for this reason, the dimension of SSG is more longitudinal to create more chances for players to play with a striker. The other important point in this scenario is that one player played 30 minutes and it was his first match after a grade 2 hamstring muscle injury and logically the training needs to be modified for him not to overload hamstring muscles that cannot recover fast. In this example, if a young player with a good training background and high fitness level has played 30 minutes, he could finish training with all its items. At the end of this session, the volume of HSR in HIIT can be adjusted and individualized according to each player’s position or match profile by manipulating the repetitions.

In scenario B (Table 1), there is a congested schedule and the match day is MD-3 for the next match. As it is not logical to supplement MD+1 (which is also MD-2) with HIIT in the end, to avoid accumulated fatigue close to the next match, the best way is to split HIIT and SSG across two days. In this scenario (B) the HIIT encompasses two parts: one more aerobic type including 1 x 8 reps of 15’’15’’ with 90% of V_IFT_ and then another type of neuromuscular HIIT including 1 x 8 reps of 10’’-20’’ with 110% of V_IFT_. The important point here is that a player who has played 40 minutes has done a high number of sprints and accumulated about 50% of his individual match profile in HSR and there is no need to overstress his hamstring muscles further with running-based HIIT In this scenario the manager’s philosophy is fast playing and quick transitions and the strength and conditioning coach will use two rules to increase the intensity of 3 v 3+Gk in MD-2. One is the touching limitation (maximum three touches) that pushes players to make fast decisions and the other rule is that they must pass the middle line to verify their scored goals, which causes a fast transition from defence to attack.

In Scenario C (Table 1), MD+1 is off (decision of the manager) and the game is at home, so post-match training in the stadium is possible. In this scenario because the players do not have a game at the end of the session and the day after is off, the type of HIIT is not split into two sets and players complete one long set of 6 min (12 reps) of 10’’-20’’ with 110% of V_IFT_. Of course, in MD+2 they will train more (mostly with the ball) than starters to achieve a greater load to compensate. In this scenario (C, Table 1), the philosophy of the coach is to have more ball possession and play with wingers and the strength and conditioning coach uses the rules of points for the number of passes and organizes the pitch with more width (double box, 40 x 33 m) to create more chances to play with wingers in MSG (5V5+Gk). In this scenario as players accumulated a high volume of HSR on MD with HIIT and MD+2 is also MD-3, there is no need to supplement the training of MD+2 with running-based HIIT.

## CONCLUSIONS

The current narrative review aimed to summarize studies covering compensatory training in soccer, as well as those addressing differences in training and match demands between starters and nonstarters. While evidence suggests that non-starters experience significantly lower loads in critical aspects such as total distance, high-speed running, or sprinting, the impact of being a starter or non-starter on physical fitness parameters is not clearly observable. Research on compensatory training for non-starters is predominantly descriptive, focusing on practices and acute effects. There is a visible gap in experimental studies examining the effects of compensatory training in soccer players, and comparative approaches (e.g., training stimulus, volume, individualization vs. generalization) are lacking, hindering practitioners’ ability to make informed decisions.

Although compensatory training has been established in strength and conditioning practices in soccer, the research gap prevents definitive support for decisions in this area. Therefore, substantial investment in both research and practice is necessary, as current approaches rely on trial and error. Caution is advised, and making definitive statements or recommendations is not advisable. Individualized intervention and careful consideration are recommended for compensatory training, while the scientific community should prioritize the development of research initiatives to inform future decisions in this field.
